# Relationship between the Engel Coefficient, Life Satisfaction, and Subjective Health for Senior Citizens in Korea: Moderating Effect of COVID-19

**DOI:** 10.3390/bs13010022

**Published:** 2022-12-26

**Authors:** Joonho Moon, Jinsoo Hwang, Won Seok Lee

**Affiliations:** 1 Department of Tourism Administration, Kangwon National University, Chuncheon 24341, Republic of Korea; 2 Department of food Service Management, Sejong University, Seoul 05006, Republic of Korea; 3 Department of Tourism and Recreation, Kyonggi University, Suwon 16227, Republic of Korea

**Keywords:** Korean senior citizen, subjective health, life satisfaction, Engel coefficient, COVID-19

## Abstract

The purpose of this study was to explore the determinants of life satisfaction for Korean senior citizens. Subjective health and the Engel coefficient were used as the explanatory variables. This research also aimed to examine the moderating effect of Coronavirus disease 2019 (COVID-19) on the relationship between the Engel coefficient and life satisfaction for Korean senior citizens. Archival data (Korean longitudinal study of aging) were used for this work. The study period is 2018 and 2020; the number of observations was 3879. Multiple linear regression analysis was performed to test the research hypotheses. To examine further, this research performed additional analyses: sub-sample analysis, median split, and independence tests. The results indicated that the Engel coefficient is negatively associated with life satisfaction, while subjective health is positively related to life satisfaction. Moreover, Korean senior citizens’ life satisfaction was higher during the COVID-19 pandemic. This research provides information for building policy for senior Korean citizens.

## 1. Introduction

Ageing has become a serious issue in Korean society. According to Statistics Korea [1], 16.1% of the Korean population is older than 65 years old. Statistics Korea [1] also anticipated that the proportion of the population older than 65 years will reach 35.3% in 2040. Thus, ageing is a crucial issue for Korean society. To cope with ageing problems, investigation of the elderly is a logical starting point, and it is therefore worthwhile to inspect the characteristics of senior citizens.

The main attributes of this research are life satisfaction and subjective health. Previous studies have stated that both life satisfaction and subjective health are critical indicators for measuring an individual’s status [2,3]. The importance of these indicators has encouraged many scholars to implement research using life satisfaction [4,5,6] as the central elements. Fertile studies also unveiled that subjective health is a critical antecedent of life satisfaction [7,8,9,10]. Given the large body of work on these factors, this research is to examine the causal effect between subjective health and life satisfaction.

Another main element of this study is the Engel coefficient, which is the proportion of food-related expenditure out of total living expenditure [11,12]. Scholars contend that a high Engel coefficient lowers living standards because higher food expenditure constrains resources to invest in other areas to improve life satisfaction [13,14]. Namely, a higher Engel coefficient causes lower individual life satisfaction. Despite such an argument of the Engel coefficient, scant studies have empirically demonstrated the effect of the Engel coefficient on the living conditions of senior citizens. Hence, this research selects the Engel coefficient as the main explanatory attribute to affiliate the research void. Since the ageing trend of Korea has become more vital, it is valuable to perform research to ascertain the impact of the Engel coefficient on senior citizens’ life satisfaction.

Coronavirus disease 2019 (COVID-19) is also considered in the present work. Prior studies have pointed out that COVID-19 has changed daily life because of restrictions due to the risk of infection [15,16,17,18]. In this context, this study seeks to examine how COVID-19 has changed individuals’ behavior. That is, the behavioral patterns between pre-COVID 19 and the COVID-19 pandemic are likely to appear as varied. This suggests that COVID-19 is likely to exert a moderating effect. Thus, this research selected COVID-19 as the moderating variable by performing a longitudinal study. Next, this research used the Korean longitudinal study of aging (KLOSA) as the source of the data. The extant literature has employed KLOSA to scrutinize the characteristics of senior citizens [19,20,21]. Such popularity ensures the credibility of the data for statistical analysis, which leads this work to select KLOSA as the main source of information.

Overall, this research attempts to (1) investigate the association between subjective health and life satisfaction, (2) examine the impact of the Engel coefficient on life satisfaction, and (3) attest the moderating effect of COVID-19 on the relationship between the Engel coefficient and life satisfaction using Korean senior citizens as the main research target. This study contributes to the literature by empirically clarifying the link between life satisfaction, subjective health, the Engel coefficient, and COVID-19. Using the outcome of this work, this study proposes implications to establish more adequate policies for the elderly.

## 2. Literature Review and Hypothesis Development

### 2.1. Life Satisfaction

Life satisfaction is how a person assesses their overall living condition; a higher level of life satisfaction indicates an individual’s better condition for living [3,4,5]. Life satisfaction has been commonly used as the outcome variable in previous studies because it represents a person’s evaluation of their current life condition [3,6]. As an example, Orben et al. [22] performed research on adolescents, employing life satisfaction as a dependent variable. An et al. [23] also inspected the determinants of life satisfaction among Korean middle- and old-aged people. Moreover, many studies of older adults have adopted life satisfaction as an explained variable [2,6,24,25]. It can be inferred that life satisfaction is a core element in research on older adults.

### 2.2. Subjective Health and Life Satisfaction

Subjective health refers to an individual’s subjective appraisal of their health condition both mentally and physically [7,8]. Health is a prerequisite for quality of life because health enables individuals to experience diverse activities: leisure, sports, economic activity, gathering, etc. [9,10]. Korff and Biemann [26] alluded that subjective health is a crucial prerequisite for satisfactory life because losing health restricts overall daily life. In fact, a vast body of research has disclosed the association between subjective health and life satisfaction. For instance, Fukahori et al. [27] showed that life satisfaction of middle-aged people is positively influenced by subjective health. Stephan et al. [2] also revealed a positive impact of subjective health on life satisfaction by choosing older adults as research subjects. In a similar vein, Gwozdz and Sousa-Poza [28] researched older German adults, and the finding indicates that subjective health is an essential element to elevate the level of life satisfaction. Matud et al. [29] also demonstrated the positive impact of subjective health on life satisfaction by examining Spanish elderly people. Ko and Jung [3] uncovered the positive effect of subjective health on life satisfaction by examining Korean older adults. Similarly, Park et al. [30] found a positive association between life satisfaction and subjective health by implementing structural equation modeling approach. Given the fertile evidence from the literature, this research proposes the following research hypothesis:

**Hypothesis** **H1:** *Life satisfaction is positively influenced by subjective health.*

### 2.3. Engel Coefficient

The Engel coefficient is the proportion of food expenditure out of all living expenditure [11,12,14]. A high Engel coefficient indicates lower quality of life because an insufficient budget is available for other areas of life: hobbies, education, clothes, etc. [12,13,31]. Thus, an individual’s needs for mental health and life quality are less likely to be satisfied with a high Engel coefficient. Previous works have discussed the Engel coefficient. Oh and Kim [32] found that a high Engel coefficient lowers life satisfaction. Qin et al. [33] examined the Chinese case, and their findings suggested that a high Engel coefficient is negatively associated with life satisfaction. Hence, it can be inferred that a higher Engel coefficient is likely to exert a negative impact on human life satisfaction. However, insufficient empirical works have explored the effect of the Engel coefficient on human life in the area of older adults. Considering the extant literature, this research thus proposes the following research hypotheses:

**Hypothesis** **H2:** *The Engel coefficient negatively affects life satisfaction.*

### 2.4. COVID-19

COVID-19 has greatly affected daily life patterns by minimizing contact with others [16,18]. Regarding food consumption patterns, people eat out less, and food delivery services have become more popular in the market [15,17,34]. Additionally, decreased interaction among people causes isolation, which in turn results in a reduction in indoor social activities: sports, art, gatherings, etc. [17,35,36,37]. Lee and You [38] also observed that the frequency of social events was rapidly decreased during COVID-19 in Korea. Plus, Lee et al. [39] observed that Korean elderly people’s mental status was devastated during COVID-19 due to isolation and illiteracy of using technology for communication. Suh et al. [40] reported that the depression of Korean older adults became more severe during COVID-19 because they lost their working place. Such a life pattern might be likely to impact happiness perception because people are constrained in their ability to exercise and engage in hobbies [41,42]. As indirect evidence, prior research revealed that people have been suffering from depression and loneliness since COVID-19, which worsens the overall life condition of individuals [35,36,37]. Considering the abovementioned aspects, the effect of food consumption during COVID-19 is likely to vary compared to that pre-COVID-19. Due to COVID-19, the financial condition of senior citizens might be likely to be worse, which causes more financial burden. Such a financially constrained condition might be likely to make life condition of older adults worse. Given the review of the literature, this research proposes the following research hypotheses:

**Hypothesis** **H3:** *COVID-19 exerts a significant negative moderating effect on the association between the Engel coefficient and life satisfaction*.

## 3. Method

### 3.1. Research Model and Data Collection

**Figure 1:**
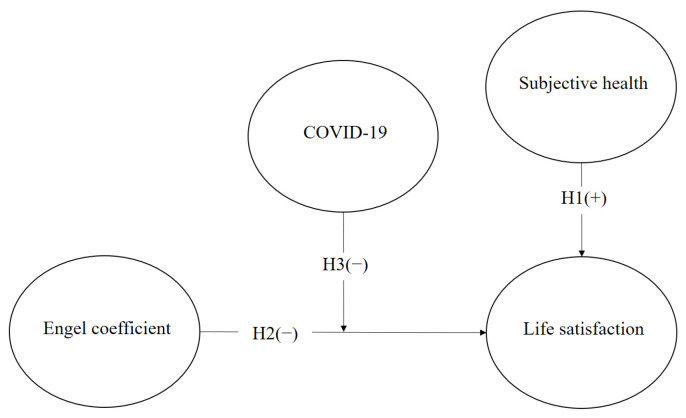
Research model.

Figure 1 shows the research model. The independent attributes of this research are subjective health (SHE) and the Engel coefficient (ENG). The explained variable of this work is life satisfaction (LSA). Additionally, this research uses COVID-19 (COV) as the moderating variable. Moreover, SHE positively affects LSA; ENG is negatively related to LSA.

**Figure 2:**
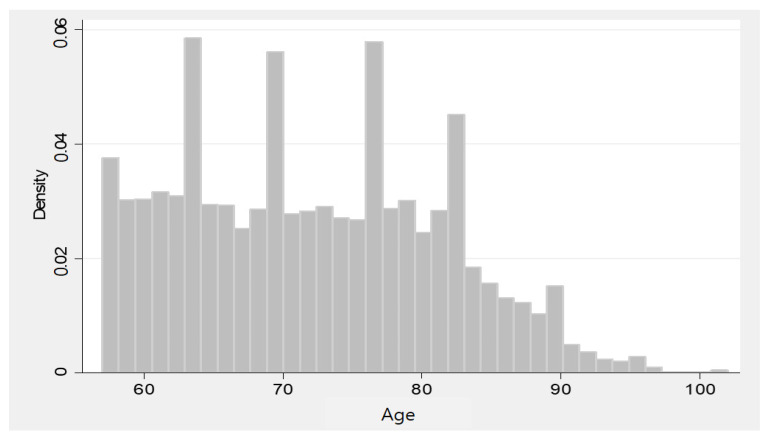
Age-information histogram.

This study obtained the data using archival sources from the KLOSA. Many studies have adopted KLOSA as the source of information for studying senior citizens [19,20,21,43]. This fact implies that the data are credible for statistical inference. The Korea Employment Information Service performed a longitudinal study of the ageing project every two years; the organization reported the data every two years. The data collection was performed using surveys; computer-based, face-to-face interview was used for the data collection because it helps participants to control technology. The survey participant age criterion was older than 45 years old. Figure 2 illustrates the distribution of age information. The study period of this study is 2018–2020, which covers both the pre-COVID-19 and COVID-19 periods as the most updated information. Initially, this research implemented data cleaning; missing responses and refusal to respond were deleted from the sample. Thus, this study adopted 3879 (2018: 2011, 2020: 1868) observations for the data analysis. That is, the data appeared as an unbalanced panel in which the survey participants for both periods were not matched perfectly [44].

### 3.2. Illustration of Variables and Data Analysis

Table 1 describes the measurement of variables. The explained variable of this research is LSA. This study measured LSA using the average of five items, and its range is 0–100. The five items are the status of health condition, economic condition, relationship with spouse, relationship with children, and overall satisfaction with their life. SHE was measured by a five-point scale (1 = very bad, 5 = very good). This study computed the Engel coefficient (ENG) by [(Food expenditure + Eating out expenditure)/Living expenditure] × 100. Binary variables were used for the measurement of COV (0 = participants in 2018, 1 = participants in 2020) and gender (GEN) (0 = Male, 1 = Female). The measurement of age (AGE) was the physical age, and personal assets (AST) was the total assets.

For the data analysis, this research computed the mean, standard deviation (SD), minimum, and maximum values. Additionally, this research executed correlation matrix analysis to scan the overall relationship between variables. Then, this research performed multiple linear regression analysis. For the first model, the dependent variable was LSA; explanatory elements included both SHE and ENG. For model 1, COV was the moderator; this study generated an interaction variable (ENG × COV) to test the moderating effect of COV [45,46]. GEN, AGE, and AST were the control variables of this work because previous research argues that the life pattern of elderly people could vary by age, gender, and personal assets [21,31,47,48,49]. To examine the moderating effect further, this research calculated mean values based on the criteria of ENG and COV using LSA and SHE as dependent variables. This study also implemented median split analysis to examine the moderating effect of COV. A median split for ENG was performed using a median value of 28 for further analysis (0 = low-ENG group, 1 = high-ENG group), using LSA as the explained attribute. Additionally, this research performed sub-sample analysis for two study periods to scrutinize the difference in the effect of the ENG on LSA. All factors considered, this research presents the following regression equation:

*LSA_i_* = *β*_0_ + *β*_1_
*SHE_i_* + *β*_2_
*ENG_i_* + *β*_3_
*COV_i_* + *β*_4_
*ENG* × *COV_i_* + *β*_5_
*GEN_i_* + *β*_6_
*AGE_i_* + *β*_7_
*AST_i_* + *e_i_* … (1)

where *i* is the *i*th participant, *e* is the residual, *LSA* is life satisfaction, *SHE* is subjective health, *ENG* is Engel coefficient, *COV* is COVID-19, *GEN* is gender, *AGE* is physical age, and *AST* is personal assets.

## 4. Results

### 4.1. Descriptive Statistics and Correlation Matrix

Table 2 presents the results of descriptive statistics. The descriptive information of LSA (Mean = 64.66, SD = 12.68) and SHE (Mean = 2.90, SD = 0.85) is presented. The mean value of ENG is 34.96, and its standard deviation is 12.02. The mean values of COV and GEN are 0.49 (SD = 0.49) and 0.35 (SD = 0.47), respectively. Table 2 also presents the information on AGE (Mean = 72.09, SD = 9.20) and AST (Mean = 31,218.51, SD = 42,186.18).

Table 3 describes the correlation matrix. LSA positively correlates with SHE (r = 0.282), COV (r = 0.039), GEN (r = 0.042), and AST (r = 0.219). However, LSA negatively correlates with ENG (r = −0.116) and AGE (r = −0.184). SHE positively correlates with GEN (r = 0.100) and AST (r = 0.102), while SHE negatively correlates with AGE (r = −0.395). ENG positively correlates with COV (r = 0.085) and AGE (r = 0.052) and negatively correlates with AST (r = −0.078). GEN negatively correlates with AGE (r = −0.029). Moreover, AST positively correlates with GEN (r = 0.028) and AGE (r = 0.048).

### 4.2. Results of Hypothesis Testing

Table 4 shows the results of the multiple linear regression analysis. The dependent variable of Model 1 is LSA. Model is statistically significant based on F-values (*p* < 0.05). LSA was positively influenced by SHE (β = 3.589, *p* < 0.05). ENG (β = −0.100, *p* < 0.05) showed a negative and significant effect on LSA. COV (β = 2.740, *p* < 0.05) was positively associated with LSA. AGE (β = −0.146, *p* < 0.05) negatively affected LSA, whereas AST positively impacted on LSA (β = −0.001, *p* < 0.05).

Table 5 shows the results of sub-sample analysis. All four models are statistically significant regarding F-values (*p* < 0.05). The results in Models 2 and 3 show that ENG in 2018 (β = −0.095, *p* < 0.05) and ENG in 2020 (β = −0.119, *p* < 0.05) exerted a negative effect on LSA, respectively. This suggests that the effect of ENG was stronger on LSA during COVID-19 pandemic. In contrast, SHE was positively associated with LSA in both periods (2018: (β = 5.124, *p* < 0.05), 2020: (β = 1.986, *p* < 0.05). However, regarding the non-significance of ENG × COV, the difference in magnitude is not statistically significant.

**Figure 3:**
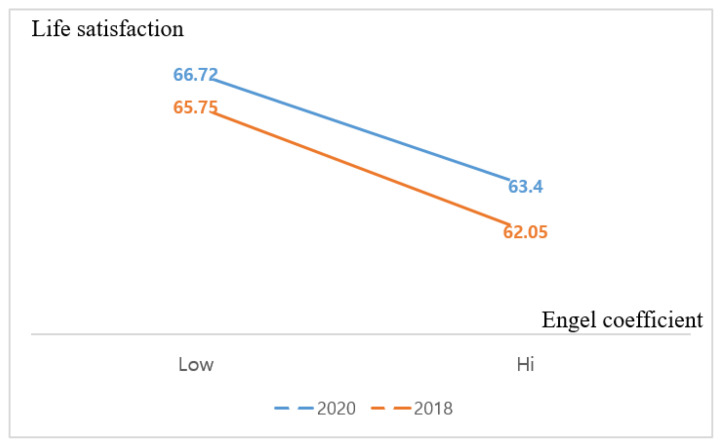
Moderating effect of COVID-19 between life satisfaction and Engel coefficient.

To scrutinize the moderating effect of COVID-19, this research presents the mean values of LSA and SHE. A median split for the Engel coefficient was implemented, and the median of the Engel coefficient was 28. Table 6 depicts the mean values of LSA (Low case in 2020 = 66.72, Low case in 2018 = 65.75, High case in 2020 = 63.40, High case in 2018 = 62.05). Figure 3 illustrate the results of the mean value calculation.

Table 7 presents the information. During the COVID-19 pandemic, improved life satisfaction of Korean older adults was ensured (*p* < 0.05). This could be explained by the reduced number of observations. That is, senior citizens with poor life satisfaction could not keep participating in the survey after COVID-19. Plus, the results reported that the Engel coefficient increased after the outbreak of COVID-19 (*p* < 0.05).

## 5. Discussion and Conclusions

This research mainly examined the moderating effect of COVID-19 on the relationship between the Engel coefficient and life satisfaction. Additionally, this study investigated the association between subjective health and life satisfaction. The results implied that subjective health is an essential precondition for a higher level of life satisfaction in the case of Korean older adults. The results are aligned with the finding of Matud et al. [29] and Ko and Jung [3] in that subjective health is positively associated with life satisfaction for the elderly. Additionally, the results demonstrated that the Engel coefficients of Korean senior citizens lowered life satisfaction. This is because budgets could be constrained more to other areas (e.g., leisure, recreation, self-development, etc.) for enhancing life condition. The finding is externally validated by the results of prior studies [32,33].

Regarding the findings, the results disclosed that the life satisfaction of the senior citizens became better during the COVID-19 pandemic. This might be due to the loss of survey participants having lower level of life satisfaction. As elderly people with poor life condition are a vulnerable population with worse chances of survival under pandemic conditions of fatal infectious disease, the results are contradictory with the findings of the extant literature [49,50]. In detail, Zhang et al. [49] and Ferreira et al. [50] researched survey participants whose survival is less endangered by COVID-19; the results showed that their overall life satisfaction was lowered during the period of COVID-19.

In contrast, the results revealed that the moderating effect of COVID-19 was not significant. However, in the sub-sample analysis, it is found that the magnitude of the Engel coefficient during COVID-19 was stronger as compared to pre-COVID 19. It can be inferred that survey participants’ lives became worse during the COVID-19 due to food expense. This might be due to the decreased working place by diminishing face-to-face transaction. It is also possible that senior citizens are relatively inactive compared to younger generations, which might exert a non-significant moderating effect of COVID-19.

Furthermore, the results considering control variables also showed that age negatively affected life satisfaction; personal assets exerted a positive impact on life satisfaction. It can be inferred that older senior citizens’ life condition became worse, as well as possessing wealth being imperative for better life in old age.

## 6. Conclusions

### 6.1. Theoretical and Practical Implications

There are theoretical contributions of this research. Above all, this research contributes to the literature by scrutinizing the Engel coefficients on senior citizens’ life satisfaction. Even though the Engel coefficient is a popular term, sparse empirical studies have been conducted to account for the behavioral characteristics of senior citizens. By addressing this research gap, this study contributes to the literature in the Engel coefficient research domain. Additionally, this study presents external validity for the relationship between subjective health and life satisfaction. The results are aligned with the findings of prior studies on the impact of subjective health on life satisfaction [2,3,27].

This study has implications for policy design. First, the government might need to dedicate budgetary resources to health-related areas because subjective health brings about a higher level of life satisfaction. These areas could include building outdoor exercise facilities as well as investing more in quarantine aids for indoor exercise. Moreover, policymakers could consider offering subsidies or food coupons for food expenditure. Such support could reduce the Engel coefficient; senior citizens could spend their money on more varied domains to take care of their needs. Conversely, the government could provide financial support for food sellers, which would indirectly reduce the Engel coefficient by decreasing the price of food products. Plus, policy makers might need to contemplate budgeting for older people as well as financially poor older people because their level of life satisfaction is lower. By allocating a government budget for poor and older senior citizens, resources could be used more efficiently.

### 6.2. Limitations

This study has limitations. The dependent variable of this work was only life satisfaction. Future research might consider more diverse elements as dependent variables, such as subjective social status and depression. Such an effort might enable future research to understand the characteristics of senior citizens more deeply. Moreover, the sample of this research was restricted to senior citizens in Korea. Future research could diversify the study sample, including participants from other geographic areas as well as younger participants, because social conditions for welfare toward the elderly could vary depending on nations’ policies and levels of economic development. The abovementioned aspects could be fertile avenues for further research.

## Figures and Tables

**Table 1:** Variable description.

Name	Code	Description
Life satisfaction	LSA	(0 = Very poor, 100 = Very good)
Subjective health	SHE	(1 = Very bad, 5 = Very good)
Engel coefficient	ENG	[(Food expenditure + Eating out expenditure)/Living expenditure] × 100
COVID-19	COV	(0 = participants in 2018, 1 = participants in 2020)
Gender	GEN	(0 = Male, 1 = Female)
Age	AGE	Physical age of survey participants
Personal assets	AST	Personal assets (10,000 KRW)

Note: expenditure unit is 10,000 KRW, KRW denotes Korean won, COVID-19 stands for coronavirus 2019 disease.

**Table 2:** Descriptive statistics (*n* = 3879).

Variable	Mean	SD	Minimum	Maximum
LSA	64.64	12.68	4	100
SHE	2.9	0.85	1	5
ENG	34.96	12.02	0	83.33
COV	0.49	0.49	0	1
GEN	0.35	0.47	0	1
AGE	72.09	9.2	57	102
AST	31,218.51	42,186.18	0	818,000

Note: SD denotes standard deviation. LSA: life satisfaction, SHE: subjective health, ENG: Engel coefficient. COV: COVID-19, GEN: gender, AGE: physical age, and AST: personal assets.

**Table 3:** Correlation matrix (*n* = 3879).

Variable	1	2	3	4	5	6
1. LSA	1					
2. SHE	0.282 *	1				
3. ENG	−0.116 *	−0.007	1			
4. COV	0.039 *	−0.012	0.085 *	1		
5. GEN	0.042 *	0.100 *	0.006	0.181 *	1	
6. AGE	−0.184 *	−0.395 *	0.052 *	0.105 *	−0.029 *	1
7. AST	0.219 *	0.102 *	−0.078 *	0.028 *	0.048 *	−0.080 *

Note: * *p* < 0.05. LSA: life satisfaction, SHE: subjective health, ENG: Engel coefficient. COV: COVID-19, GEN: gender, AGE: physical age, and AST: personal assets.

**Table 4:** Results of hypotheses testing (*n* = 3879).

Variable	Model1 β (t-Stat)
Intercept	64.418 (29.87) *
SHE	3.589 (14.37) *
ENG	−0.100 (−4.26) *
COV	2.740 (2.31) *
ENG × COV	−0.019 (−0.60)
GEN	0.625 (1.61)
AGE	−0.146 (−6.09) *
AST	0.001 (12.62) *
F-value	91.57 *
R^2^	0.1421

Note: * *p* < 0.05, Dependent variable: LSA in model 1, LSA: life satisfaction, SHE: subjective health, ENG: Engel coefficient. COV: COVID-19, GEN: gender, AGE: physical age, and AST: personal assets.

**Table 5:** Results of hypotheses testing: subsample analysis (N_2018_ = 2011; N_2020_ = 1868).

Variable	Model 2 2018 β (t-Stat)	Model 3 2020 β (t-Stat)
Intercept	65.330 (23.12) *	66.914 (20.53) *
SHE	5.124 (15.26) *	1.986 (5.44) *
ENG	−0.095 (−4.23) *	−0.119 (−5.12) *
GEN	1.774 (3.26) *	0.219 (0.39)
AGE	−0.231 (−6.94) *	−0.080 (−2.30) *
AST	0.001 (7.32) *	0.001 (10.35) *
F-value	110.08 *	40.84 *
R^2^	0.2154	0.1421

Note: * *p* < 0.05, Dependent variable: LSA in Models 2 and 3, LSA: life satisfaction, SHE: subjective health, ENG: Engel coefficient. COV: COVID-19, GEN: gender, AGE: physical age, and AST: personal assets.

**Table 6:** Mean values for moderating effect of COVID-19 (*n* = 3879).

LSA	Low ENG	Hi ENG
2020	66.72	63.40
2018	65.75	62.05

Note: Median Engel coefficient 28, LSA: Life satisfaction, ENG: Engel coefficient.

**Table 7:** Results of independent *t*-test regarding COVID-19.

Variable	Pre-COVID-19 Mean (SD) (*n* = 2005)	Post-COVID-19 Mean (SD) (*n* = 1874)	*t*-Value
LSA	64.16 (12.65)	65.16 (12.70)	−2.452 *
ENG	33.95 (11.63)	36.00 (12.33)	−7.436 *

Note: * *p* < 0.05, SD stands for standard deviation. LSA: Life satisfaction, ENG: Engel coefficient.

## Data Availability

Not applicable.
